# External Quality Assessment for the Detection of Measles Virus by Reverse Transcription-PCR Using Armored RNA

**DOI:** 10.1371/journal.pone.0134681

**Published:** 2015-08-05

**Authors:** Dong Zhang, Yu Sun, Tingting Jia, Lei Zhang, Guojing Wang, Rui Zhang, Kuo Zhang, Guigao Lin, Jiehong Xie, Lunan Wang, Jinming Li

**Affiliations:** 1 National Center for Clinical Laboratories, Beijing Hospital, Beijing, People’s Republic of China; 2 Graduate School, Chinese Academy of Medical Sciences and Peking Union Medical College, Beijing, People’s Republic of China; 3 Graduate School, Peking University Health Science Center, Beijing, People’s Republic of China; United States Army Medical Research Institute for Infectious Disease, UNITED STATES

## Abstract

In recent years, nucleic acid tests for detection of measles virus RNA have been widely applied in laboratories belonging to the measles surveillance system of China. An external quality assessment program was established by the National Center for Clinical Laboratories to evaluate the performance of nucleic acid tests for measles virus. The external quality assessment panel, which consisted of 10 specimens, was prepared using armored RNAs, complex of noninfectious MS2 bacteriophage coat proteins encapsulated RNA of measles virus, as measles virus surrogate controls. Conserved sequences amplified from a circulating measles virus strain or from a vaccine strain were encapsulated into these armored RNAs. Forty-one participating laboratories from 15 provinces, municipalities, or autonomous regions that currently conduct molecular detection of measles virus enrolled in the external quality assessment program, including 40 measles surveillance system laboratories and one diagnostic reagent manufacturer. Forty laboratories used commercial reverse transcription-quantitative PCR kits, with only one laboratory applying a conventional PCR method developed in-house. The results indicated that most of the participants (38/41, 92.7%) were able to accurately detect the panel with 100% sensitivity and 100% specificity. Although a wide range of commercially available kits for nucleic acid extraction and reverse transcription polymerase chain reaction were used by the participants, only two false-negative results and one false-positive result were generated; these were generated by three separate laboratories. Both false-negative results were obtained with tests performed on specimens with the lowest concentration (1.2 × 10^4^ genomic equivalents/mL). In addition, all 18 participants from Beijing achieved 100% sensitivity and 100% specificity. Overall, we conclude that the majority of the laboratories evaluated have reliable diagnostic capacities for the detection of measles virus.

## Introduction

*Measles virus* (MeV) is a single-stranded, negative-sense RNA virus that belongs to the genus *Morbillivirus* in the family *Paramyxoviridae* [1]. The acute and highly contagious infectious disease caused by MeV can affect multi-organ systems and lead to serious complications or even death. Therefore, although measles is immunization-preventable, it remains a threat to non-vaccinated children and adults.

Since 1986, a two-dose, routine measles immunization schedule has been used in China, with the goal of eliminating measles. Coverage of the measles vaccine was estimated to be over 90% since 2009; correspondingly, measles morbidity and mortality have dramatically declined [2,3]. An outbreak of measles (23 people infected) in Beijing in early 2015 served as a reminder that measles remains a significant health threat in China [4], despite the country’s aim to eliminate measles by 2012 [5]. Certain challenges to measles eradication still exist within the large population of migrants to urban areas and among children living in remote areas in China. Additionally, measles patients do not usually seek health care; thus, these cases are not reported. As a result, China contributes greatly to the heavy burden of measles in the WHO Western Pacific Region [6], highlighting the importance of an effective surveillance system and enhanced vaccination strategies. Sensitive and specific assays can greatly enhance the implementation of measles surveillance.

Laboratory detection of MeV can be achieved either by serological methods or by viral genome detection. Between them, reverse transcription polymerase chain reaction (RT-PCR) methodology has served as an indispensable tool for laboratory detection of acute infection [7, 8]. This preference for RT-PCR is mainly because of the reduced positive predictive value of serological detection (typically enzyme immunoassays (EIAs) for measles-specific immunoglobulin M (IgM) antibodies) in areas with low prevalence [9]. In 2013, laboratories belonging to the measles surveillance system (MSS) of China initiated the use of nucleic acid tests (NATs) to detect MeV, as per the requirements specified by the latest National Measles Surveillance Guideline. Although these laboratories may have previously conducted NATs to detect other pathogens for years, little was known about the performance of these laboratories in routine MeV detection, especially that of laboratories located in megacities containing large proportions of China’s floating population, such as Beijing and Shanghai.

In this pilot study, a nationwide external quality assessment (EQA) was carried out by the National Center for Clinical Laboratories (NCCL) of China to provide information about the measles diagnosis proficiency of laboratories around mainland China.

## Materials and Methods

### Sample preparation

To calibrate the number of MeVC RNAs and MeVV RNAs to an international unit (IU) value, we designed two 1,002-bp chimeric sequences to include the HCV 5’UTR region (a 368-bp target sequence amplified from pNCCL-HCV archived in our laboratory [10]) and RT-PCR targets. Specimens of circulating MeV strain MVi/Zhejiang.CHN/7.05/4 (MeVC) (GenBank accession no. DQ211902.1) and MeV vaccine strain Shanghai-191 (MeVV) (GenBank accession no. FJ416067.1) were kindly provided by the Beijing Center for Diseases Prevention and Control. The chimeric sequences were generated by overlapping extension PCR (primer sequences are listed in S1 Table). Subsequently, the chimeric sequences were inserted into pACYC-MS2 [11], which were constructed with three modified stem-loop (pac site) of MS2. Gel-purified overlapping extension PCR products were subcloned into the *Pac*I and *Nde*I sites of the pACYC-MS2 vector. The recombinant plasmids were transformed into *E*. *coli* strain BL21 (DE3) to express armored RNAs according to previously published [12, 13].

After RNase and DNase treatments, armored RNAs were harvested using conventional purification methods [14, 15]. Finally, the purified armored RNAs were calibrated against the World Health Organization (WHO) Second International Standard for HCV RNA (National Institute for Biological Standards and Controls [NIBSC], code 96/798, UK), using an HCV RNA PCR fluorescence quantitative diagnostic kit (Shanghai Kehua Bio-engineering Co., Ltd., Shanghai, China), and the ratio of HCV RNA copies to international units was 1:1 according to the technical manual.

### Panel composition

The panel consisted of 10 coded samples: seven positive samples, containing armored RNAs at concentrations ranging from 1.2 × 10^4^ genomic equivalents/mL to 5.6 × 10^5^ genomic equivalents/mL, and three negative samples. The positive samples were obtained by appropriately diluting armored RNAs. These included one specimen of MeVV with a concentration of 1.2 × 10^4^ genomic equivalents/mL, which was prepared to test the laboratories’ ability to detect MeV strains other than the prevalent strains, as well as to determine the detection limit of each commercial kit. The positive samples also included two replicate specimens (No. 1402 and No. 1406, each with a concentration of 4 × 10^4^ genomic equivalents/mL), which were prepared to evaluate the repeatability of each participant’s procedure. Negative samples were prepared from DMEM that contained no virus (Table 1).

**Table 1:** Composition of the EQA panel and qRT-PCR results.

Sample no.	Concn. of armored RNAs in samples (genomic equivalents/mL)	Classification	No. of correct/total no. tested (%)
1401	-	Negative	41/41 (100)
1402	4 × 10^4^	MeVC	41/41 (100)
1403	5.6 × 10^5^	MeVC	41/41 (100)
1404	5.2 × 10^4^	MeVC	41/41 (100)
1405	1.7 × 10^5^	MeVC	41/41 (100)
1406	4 × 10^4^	MeVC	41/41 (100)
1407	1.2 × 10^4^	MeVV	39/41 (95.1)
1408	-	Negative	40/41 (97.6)
1409	5.6 × 10^5^	MeVC	41/41 (100)
1410	-	Negative	41/41 (100)
Total			408/410 (99.3)

### EQA organization

The test samples were shipped on ice to the participating laboratories along with the data sheets. Each participant was asked to detect MeV in the panel by following routine operating procedures and to report the results on the data sheet. Participants were asked to report information such as the qualitative results and the threshold cycle value of each sample, the RNA extraction methods and instruments used, and the time point at which each laboratory began the procedure for MeV detection. The results were returned to the NCCL via e-mail or fax within 2 weeks of receiving the sample.

The results were classified as “competent” (100% correct results), “acceptable” (≤ 2 incorrect results), or “improvable” (> 2 incorrect results). All data were analyzed using SPSS version 16.0. Sensitivities between different groups were compared using Pearson’s chi-square test or Fisher’s exact test, as appropriate. *P* values < 0.05 were considered statistically significant.

## Results

### Quality assessment of samples

To determine whether armored RNAs encapsulating target sequences were successfully expressed, we conducted 1% agarose gel electrophoresis with ethidium bromide staining and sodium dodecyl sulfate polyacrylamide gel electrophoresis (SDS-PAGE). A single band between 1 kb and 2 kb in size was visible (S1A Fig). The molecular weight of the proteins in each sample was approximately 14 kD (S1B Fig). Armored RNAs were observed by transmission electron microscopy, as shown in S1C Fig; the diameter of the MS2 virus-like particles (armored RNAs) was approximately 30 nm. In addition, RT-PCR was employed to confirm encapsulation of the target sequences. As expected, the target sequences were successfully amplified (S1D Fig), which was subsequently confirmed by sequencing (data not shown).

A quantitative assay for HCV RNA (Shanghai Kehua Bio-engineering Co., Ltd., Shanghai, China) was performed to validate the genomic equivalents of the seven positive samples containing armored RNAs; the ratio of genomic equivalents to international units was 1:1. To verify the availability of the armored RNAs for the EQA study, we also examined their stability before panel distribution. Stability analyses revealed that the armored RNAs were stable for at least 2 weeks at 37°C, 4 weeks at room temperature, and 2 months at both 4°C and -20°C (data not shown). The freshly prepared armored RNAs were incubated with DNase I and RNase A at 37°C for 60 min and subsequently analyzed by agarose gel electrophoresis (1%) with ethidium bromide staining, which demonstrated that they were completely resistant to both DNase and RNase treatments (S1A Fig).

### Distribution and reporting of the results of the panel

In August 2014, specimens were dispatched to laboratories in 15 different provinces, municipalities, or autonomous regions. Most of the laboratories (n = 18) that participated in our study were located in Beijing; the other 23 laboratories were located in Shanghai (n = 4), Tianjin (n = 1), Jiangsu (n = 2), Anhui (n = 2), Henan (n = 3), Liaoning (n = 2), Hunan (n = 2), Guangdong (n = 1), Yunnan (n = 1), Inner Mongolia (n = 1), Guangxi (n = 1), Shandong (n = 1), Jiangxi (n = 1), and Sichuan (n = 1). Among them, 40 laboratories belonged to the MSS of China, and one laboratory was a diagnostic reagent manufacturer.

In total, 41 laboratories submitted their results. All participants reported that they applied routine NATs for the diagnosis of MeV. Twenty-eight laboratories had performed routine MeV nucleic acid detection since before 2013, while the others had initiated detection in 2014.

The participants applied a range of commercial kits for nucleic acid extraction. The RNeasy Mini Kit (Hangzhou woosen biotechnology Co.Ltd., Jiangsu, China) and the QIAamp Viral RNA Mini Kit (Qiagen) were widely used by participants (27/41, 65.9%), and seven other RNA extraction kits were used by the remaining participants (14/41, 34.1%). Most participants manually extracted viral RNA, while 11 (26.8%) laboratories each used one of the following automated nucleic acid extractors: QIAcube (n = 4), MagNA Pure LC 2.0 (n = 3), TIANLONG NP968 (n = 2), TANBead (n = 1), and MagMAX Express (n = 1). All participants (n = 40) employed commercially available reverse transcription-quantitative PCR (qRT-PCR) kits for molecular detection of MeV sequences, except for one laboratory that applied a kit developed in-house. Among the laboratories’ data sets, 28 (70%) were generated using BioPerfectus kits (Jiangsu BioPerfectus Technologies Co., Ltd., Jiangsu, China), nine (22.5%) data sets were generated using Uninovo kits (Jiangsu Uninovo Biological Technology Co., Ltd, Jiangsu, China), and the remaining laboratories (n = 3) used kits from Beijing Kinghawk Pharmaceutical Co., Ltd. (Beijing, China), Daan Gene Co., Ltd. (Guangzhou, China), and Mabsky Biotech Co. Ltd. (Shenzhen, China) (Table 2).

**Table 2:** Comparison of sensitivities and specificities of different assays.

Assay		A^a^	B^a^	C^a^	D^a^	E^a^	F^b^	Total
No. of data sets		28	9	1	1	1	1	41
No. of correct results/total no. of results	1401	28/28	9/9	1/1	1/1	1/1	1/1	41/41
	1402	28/28	9/9	1/1	1/1	1/1	1/1	41/41
	1403	28/28	9/9	1/1	1/1	1/1	1/1	41/41
	1404	28/28	9/9	1/1	1/1	1/1	1/1	41/41
	1405	28/28	9/9	1/1	1/1	1/1	1/1	41/41
	1406	28/28	9/9	1/1	1/1	1/1	1/1	41/41
	1407	28/28	9/9	1/1	0/1	0/1	1/1	39/41
	1408	28/28	8/9	1/1	1/1	1/1	1/1	40/41
	1409	28/28	9/9	1/1	1/1	1/1	1/1	41/41
	1410	28/28	9/9	1/1	1/1	1/1	1/1	41/41
Sensitivity (%)		196/196 (100)	63/63 (100)	7/7 (100)	6/7 (85.7)	6/7 (85.7)	7/7 (100)	285/287 (99.3)
Specificity (%)		84/84 (100)	26/27 (96.3)	3/3 (100)	3/3 (100)	3/3 (100)	3/3 (100)	122/123 (99.2)

^a^ Five commercial TaqMan real-time RT-PCR kits for MeV RNA detection: A (BioPerfectus Technologies, Jiangsu, China), B (Jiangsu Uninovo Biological Technology Co. Ltd, Jiangsu, China), C (Daan Gene Co. Ltd, Guangzhou, China), D (Beijing Kinghawk Pharmaceutical Co. Ltd, Beijing, China), E (Mabsky Biotech Co. Ltd, Shenzhen, China).

^b^ F: in-house developed qRT-PCR assay for measles detection.

### Overall performance of laboratories

Of the 41 completed data sets, 38 (92.7%) analyses were found to be “competent”. Three participants (7.3%) reported one false-positive or one false-negative, and thus were classified as “acceptable”, according to the criteria. Eventually, 430 results were received, of which three (0.7%) were incorrect, including two false-negative results targeting sample No.1407; false-positive results were infrequently reported (1/126) (Table 1). Participants correctly reported the presence of MeVC in all samples even when the concentration was as low as 4 × 10^4^ genomic equivalents/mL. In clinical settings, the lower limit of MeV RNA concentration has been reported as 1.1 × 10^4^ genomic equivalents/mL [16]. Therefore, most participants (38/41, 92.7%) were classified as “competent” for routine diagnostic MeV detection. No difference in competency was noted for the detection of two pairs of duplicate specimens (1403 and 1409, with a concentration of 5.6 × 10^5^ genomic equivalents/mL; and 1402 and 1406, with a concentration of 4 × 10^4^ genomic equivalents/mL); all participants reported correct results for these specimens.

We next assessed the performances of different assays. The overall sensitivity and specificity of the commercial assays were 99.3% and 99.2%, respectively. The commercially available assay manufactured by Jiangsu BioPerfectus Technologies Co., Ltd. was the most widely used by the participating groups, and all participants that applied this assay achieved a “competent” result (Table 2). Meanwhile, two laboratories, one using a kit from Kinghawk Pharmaceutical Co. Ltd and the other a kit from Mabsky Biotech Co. Ltd, failed to detect the weakly positive specimen (No. 1407, with a concentration of 1.2 × 10^4^ genomic equivalents/mL). However, we cannot make the conclusion that the BioPerfectus assay is better than the other commercial kits because of the limited number of groups using the other commercial assays. Only one participating group reported results generated by an in-house developed assay, which were given a “competent” score.

We did not find any difference in nucleic acid extraction performance among laboratories that used different extraction kits (*P* > 0.05). We further compared the performance of automated viral RNA extraction with that of manual RNA extraction. The results showed no difference between the two extraction methods (*P* > 0.05).

## Discussion

In China, although the annual incidence of measles has decreased from 9.95 per 100,000 people in 2008 to 0.46 per 100,000 people in 2012 [17], the 2015 resurgence reminded us that more efforts are needed to eradicate measles. Detection of MeV RNA by RT-PCR is an increasingly common method of measles surveillance because of the convenience and non-invasiveness of obtaining throat or nasopharyngeal swabs. In addition, the detection of MeV RNA plays a pivotal role in the laboratory confirmation of infection [7]. Because most MSS laboratories only recently began using NATs to detect MeV, the NCCL organized this EQA program to determine whether relevant laboratories in China could provide reliable data for MeV RNA detection and to provide technical information on the currently available kits for laboratory-based measles surveillance.

In this EQA, 97.6% (40/41) laboratories used commercial kits from five different manufacturers. One laboratory used an in-house endpoint method. A plausible reason for this may be that the qRT-PCR method is usually more sensitive than a conventional RT-PCR assay. Furthermore, the endpoint assay is routinely used to amplify the region of the genome necessary for confirming the MeV genotype. The laboratory that used an in-house method correctly detected all specimens.

The panel results suggest that most of the participating laboratories (38/41, 92.7%) can accurately detect MeV RNA by using RT-PCR; only two false-negative results and one false-positive result were reported. In comparison, the overall sensitivities and specificities achieved in this MeV EQA program were better than those achieved in the NCCL’s EQA programs for human enterovirus 71 (HEV71), coxsackievirus A (CA16), and avian influenza A (H7N9) virus [3, 6]. It should be mentioned that all 18 laboratories from Beijing accurately detected the specimen (No. 1407) that had a concentration of 1.2 × 10^4^ genomic equivalents/mL with 100% specificity. Among them, nine groups used commercial kits from Jiangsu BioPerfectus Technologies Co., Ltd. and the remainder used commercial kits from Jiangsu Uninovo Biological Technology Co., Ltd. We are not surprised by this outcome because, of all the regions, Beijing was one of the first to initiate the use of MeV NATs, with all MSS laboratories from 18 districts or counties implementing routine MeV NATs in 2013. In contrast, two laboratories were unable to detect MeVV in sample No. 1407. For these two groups, greater scrutiny is required to identify the possible reasons for decreased sensitivities owing to either kit performance or laboratory operations. It should be noted that decreased sensitivity could be attributed to degradation of RNA or to PCR inhibition, if the specimens were improperly processed. However, because the commercial kits that failed to detect No. 1407 were only used by a single group, we were hindered from further evaluating this problem of differing sensitivities between commercial assays.

In this EQA, a wide variety of nucleic acid extraction kits was applied. We did not find any difference in performance among groups using different nucleic acid extraction kits. Special attention should still be paid to this key procedure, despite the presence of only one false-positive result here. For laboratories using commercial kits, false-positive results are due to cross-contamination rather than non-specific products. Laboratories that perform frequent testing also have increased risks of contamination, either from specimens or from amplicons. To minimize the chance of cross-contamination, procedures that are more stringent should be established, particularly for laboratories that manually purify nucleic acids. In addition, we noticed that a higher number of laboratories applied automatic instruments (e.g., QIAcube DNA RNA Purification Extraction System, Qiagen, and MagNA Pure LC 2.0 system, Roche) to purify nucleic acids than that observed in our previous EQA programs [14, 15]. Other participants (approximately 50%) reported that they have scheduled or have already prepared for the routine use of automated nucleic acid recovery systems. The introduction of automated platforms and methods could, to a large extent, save labor and time while providing high accuracy. However, we should bear in mind that any change in standard operating procedures (SOPs) must be validated and/or verified before implementation, particularly because some groups are concerned that automatic RNA extraction might be less efficient than manual extraction.

For the preparation of this panel, we used armored RNAs encapsulating viral genes as MeV surrogates because they are stable, RNase-resistant, and noninfectious. We have previously used the same technique for EQA studies of seasonal or highly contagious pathogens [14, 15]. It is noteworthy that some participating laboratories, including those located in Sichuan or Guangxi, are almost 2000 km away from us. These groups performed well in our study, which provided circumstantial evidence that the specimens were resistant to several days of exposure to relatively harsh conditions. Based on these characteristics, we are confident in our ability to organize an even larger EQA scheme that would include additional pathogens, such as rubella and mumps viruses, as well as additional participants.

In conclusion, our pilot EQA study provided encouraging results regarding the proficiency of laboratories employing MeV NATs in China. Most of the participants detected all specimens with considerable sensitivity and specificity. Nevertheless, the limited number of participants hindered us from drawing further conclusions. In the future, we also plan to introduce armored RNAs encapsulating other prevalent genotypes of MeV into our panel because genetic characterization is important to support molecular epidemiological studies and to track transmission pathways [8].

## Supporting Information

S1 Fig Identification of armored RNAs. (DOC)

S2 Fig Armored RNA packaging system. (DOC)

S1 Table Primers used in the present study. (DOC)
