# Supplementary material for: External Quality Assessment for the Detection of Measles Virus by Reverse Transcription-PCR Using Armored RNA
Source: PLoS One. 2015 Aug 5;10(8):e0134681. doi: 10.1371/journal.pone.0134681 (PMC4526687; doi:10.1371/journal.pone.0134681)
Supplement: S1 Fig — (DOC) [file pone.0134681.s001.doc]

**S1 Fig. Identification of armored RNAs.**

**
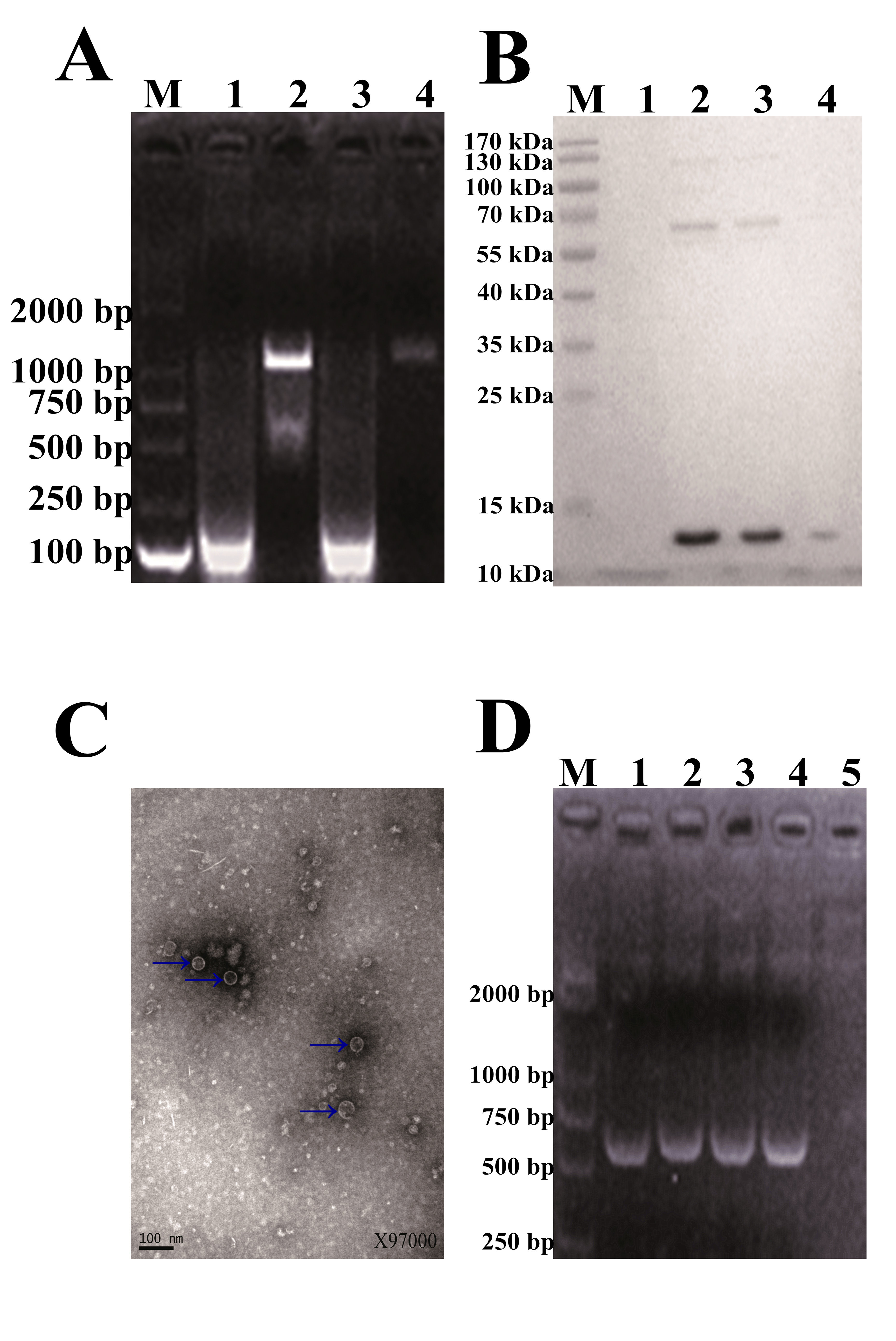
**

(A) Nuclease resistance of purified armored RNAs. Freshly prepared armored RNAs were incubated with RNase A and DNase I at 37 °C for 1 h and subsequently analyzed on a 1% agarose gel, producing bands between 1 kb and 2 kb. Lane M, molecular weight marker; Lane 1, armored RNAs of MeVC without incubation with RNase A and DNase I; Lane 2, armored RNAs of MeVC incubated with RNase A and DNase I; Lane 3, armored RNAs of MeVV without incubation with RNase A and DNase I; Lane 4, armored RNAs of MeVV incubated with RNase A and DNase I. (B) After purification by gel exclusion chromatography, freshly prepared armored RNAs were loaded onto an SDS-polyacrylamide gel, and subjected to electrophoresis in tricine buffer. Proteins were visualized by staining the gel with Coomassie brilliant blue. Lane M, PageRuler Prestained Protein Ladder; Lane 1, negative control (blank); Lane 2, positive control (MS2); Lane 3, armored RNAs of MeVC; Lane 4, armored RNAs of MeVV. (C) Identification of armored RNAs by transmission electron microscopy. The diameter of armored RNAs was approximately 30 nm. Photographs were taken with a screen magnification of 97,000 ×. (D) Ethidium bromide-stained 1% agarose gel of RT-PCR amplification products of RNA extracted from armored RNAs. Lane M, molecular weight marker; Lane 1 and 2, positive controls (PCR products using plasmids pACYC-MS2-MeVC and pACYC-MS2-MeVV as templates); Lane 3 and 4, RT-PCR products of RNA extracted from armored RNAs of MeVC and MeVV; Lane 5, negative control (blank).
