# Supplementary material for: External Quality Assessment for the Detection of Measles Virus by Reverse Transcription-PCR Using Armored RNA
Source: PLoS One. 2015 Aug 5;10(8):e0134681. doi: 10.1371/journal.pone.0134681 (PMC4526687; doi:10.1371/journal.pone.0134681)
Supplement: S2 Fig — (DOC) [file pone.0134681.s002.doc]

**S2 Fig. Armored RNA packaging system.**


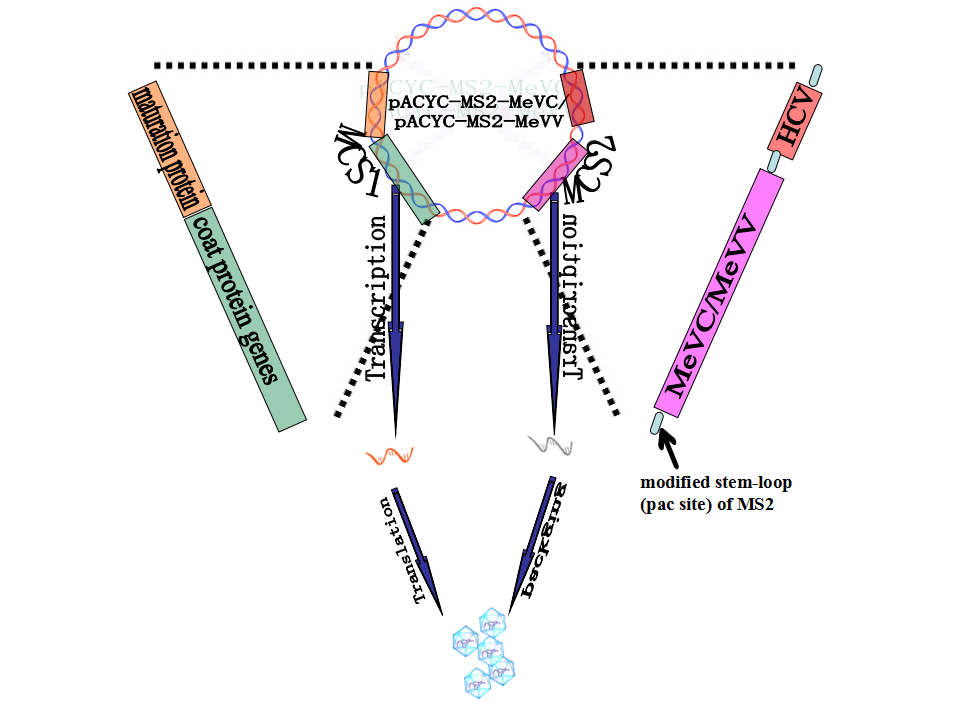


One-plasmid expression systems were constructed, in which the maturation protein and coat protein genes were expressed from MCS1 of pACYC-Deut 1 and pac sites and the chimeric RNA sequences were produced from MCS2 of pACYC-Deut 2. The pac sites were located at the both end of MeVC/MeVV and HCV, and between them. Armored RNAs were produced by inducing and expressing the one-plasmid systems.
