# Supplementary material for: External Quality Assessment for the Detection of Measles Virus by Reverse Transcription-PCR Using Armored RNA
Source: PLoS One. 2015 Aug 5;10(8):e0134681. doi: 10.1371/journal.pone.0134681 (PMC4526687; doi:10.1371/journal.pone.0134681)
Supplement: S1 Table — (DOC) [file pone.0134681.s003.doc]

**S1 Table. Primers used in the present study.**

| Primer | Sequence (5’-3’) |
| --- | --- |
| MeVF | CCCATATGACATGAGGATCACCCATGTTGGAGCTATGCCATGGGAGT |
| MeVR | GTGATCTATGGTGGAGGTACATGGGTGATCCTCATGTTAACAATGATGGAGGGTAGG |
| HCVF | TACCCTCCATCATTGTTAACATGAGGATCACCCATGTACCTCCACCATAGATCAC |
| HCVR | CCTTAATTAAACATGGGTGATCCTCATGTGGTTGGTGTTACGTTTGGTTTTTCTTTG |

Restriction sites are underlined. CATATG: *Nde* I; TTAATTAA: *Pac* I. Construction of MeVC-HCV, MeVV-HCV by overlapping PCR. During the first-round of PCR, 3 fragments (MeVC, MeVV, and the HCV 5’UTR) were separately amplified from circulating measles virus strain, measles virus vaccine, and pNCCL-HCV (constructed by our laboratory) using primers, MeVF and MeVR, as well as HCVF and HCVR, respectively. The fragments of MeVC-HCV and MeVV-HCV were subsequently obtained by a second-round PCR using primers MeVF and HCVR.
